# BODIPY-Based Ratiometric Fluorescent Probe for Sensing Peroxynitrite in Inflammatory Cells and Tissues

**DOI:** 10.3390/bios14120638

**Published:** 2024-12-22

**Authors:** Qian Wu, Ziwei Hu, Guoyang Zhang, Yulong Jin, Zhuo Wang

**Affiliations:** State Key Laboratory of Chemical Resource Engineering, College of Chemistry, Beijing University of Chemical Technology, Beijing 100029, China; ac_wuqian105@163.com (Q.W.); 15608654086@163.com (Z.H.); m17863656080@163.com (G.Z.)

**Keywords:** ratiometric probe, peroxynitrite, fluorescence imaging, inflammatory

## Abstract

Peroxynitrite (ONOO^−^) plays an important role in many physiological and pathological processes. Excessive ONOO^−^ in cells leads to oxidative stress and inflammation. However, precise monitoring of ONOO^−^ levels in specific organelles (e.g., mitochondria) is still lacking and urgently needed. Herein, we rationally designed a mitochondria-targeted ratiometric fluorescent probe, MOBDP-I, for imaging of ONOO^−^ in the mitochondria of inflammatory cells and model mice. This probe, MOBDP-I, was synthesized by conjugating a BODIPY fluorophore to a mitochondria-targeting moiety–indole-salt group by a carbon–carbon double bond (C=C). In the presence of ONOO^−^, the C=C bond between the BODIPY backbone and the indole-salt group was oxidized and broken, leading to an 18-fold enhancement of fluorescence at 510 nm, along with a significant fluorescence decrease at 596 nm. The ratiometric response property bestowed the probe with advantages in the precise quantification of ONOO^−^ in cells, thus allowing estimation of the extent of inflammation in living cells and mouse models of rheumatoid arthritis, peritonitis, and brain inflammation. MOBDP-I could act as an effective molecular tool to study the relationship between ONOO^−^ and the occurrence and development of inflammatory diseases.

## 1. Introduction

Reactive nitrogen species (RNS) are highly reactive molecules produced as metabolic byproducts in biological systems and function as important cellular signaling molecules. ONOO^−^, a type of RNS, is primarily produced in cells through the reaction of nitric oxide (NO) and the superoxide anion (O_2_^.−^) [1]. ONOO^−^ plays a critical role in regulating redox homeostasis, signal activation, transduction, and the normal immune response [2]. However, excessive ONOO^−^ production can result in various disorders, including neurological, cardiovascular, and cerebrovascular diseases, cancer, inflammatory conditions, and autoimmune diseases [3,4,5]. ONOO^−^ is mainly produced in mitochondria during drug-induced oxidative stress. Abnormal levels of ONOO^−^ cause mitochondrial depolarization, leading to oxidative damage to biomolecules and ultimately resulting in cell necrosis and apoptosis [6,7]. Inflammation is a defense mechanism against injury or infection in organisms. Oxidative stress is a key feature of inflammation, often characterized by elevated levels of reactive species during the early stages of the inflammatory response [8]. Studies have shown that ONOO^−^ is directly involved in tissue damage in patients with inflammation [9,10].

Given the high reactivity and short lifespan of ONOO^−^, traditional histological and biochemical techniques are inadequate for assessing its dynamic changes in cells or in vivo in real time. Fluorescence imaging provides a powerful tool for non-invasive research of biological processes in cells and organisms due to its superior spatial and temporal resolution, sensitivity, and ability to detect targets in real time in complex biological systems [11,12,13,14]. Currently, numerous fluorescent probes have been developed for the specific detection of ONOO^−^. The recognition mechanisms of most of probes depend on specific interactions between ONOO^−^ and certain substrates under biological conditions, such as oxidation-induced cleavage of ethylenic bonds [15], oxidation of benzoborate acid and its ester derivatives [16], oxidation-induced N-dearylation of N-aminophenol [17], oxidation of a-ketoamide derivatives [18], and oxidation of indoline-2,3-dione [19]. The recognition mechanisms not only dominate the specific reaction (reaction kinetics and sensitivity) of the probe toward ONOO^−^, but also can efficiently tune the fluorescence output performances (emission wavelengths, fluorescence quantum yields, and fluorescence lifetimes) by various fluorescence regulation principles, such as intramolecular charge transfer (ICT), photo-induced electron transfer (PET), excited-state intramolecular proton transfer (ESIPT) and Förster resonance energy transfer (FRET) [20]. BODIPY dyes have great advantages in the detection of ONOO^−^ due to their good chemical and optical stability, high quantum yield, and pH insensitivity. Zhang and colleagues developed a BODIPY-based fluorescent probe for selective detection of ONOO^−^, using phenylboronic acid pinacol ester as the recognition group [21]. Han et al. reported a small-molecule probe for monitoring ONOO^−^ levels during autophagy in cells and mice. The probe was constituted with a BODIPY fluorophore and a diphenylaniline as an ONOO^−^ recognition site [22]. Currently, there are few BODIPY-based fluorescent probes for the detection of ONOO^−^ in mitochondria, and most rely on single-signal intensity, making them susceptible to interference from measurement conditions and the cellular microenvironment. Ratiometric fluorescent probes, which provide detection results by measuring the ratio of two distinct emission intensities, have become a more reliable method for obtaining accurate qualitative and quantitative data due to their ‘self-calibration’ ability [23,24,25]. Therefore, the development of ratiometric fluorescent probes for visualizing ONOO^−^ in vivo remains a critical need.

**Scheme 1:**
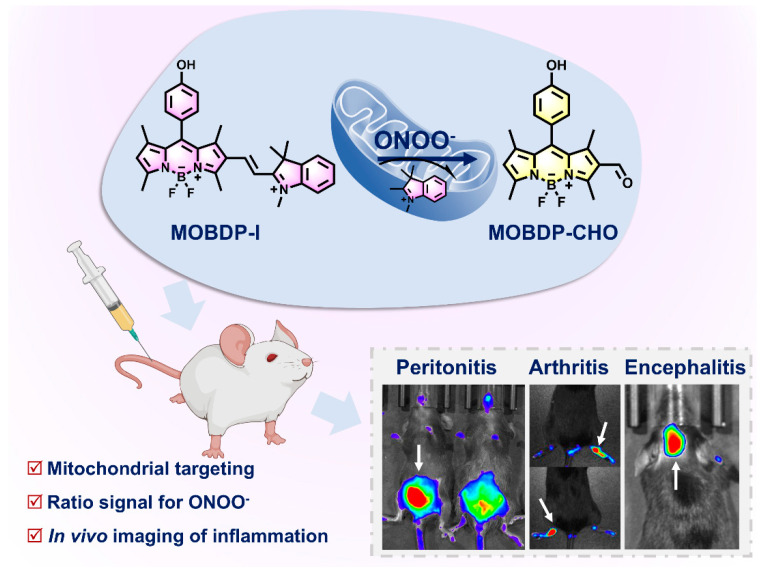
The response mechanism of MOBDP-I to ONOO^−^ and the fluorescence imaging of MOBDP-I in various inflammatory mouse models.

In this work, we designed and synthesized a mitochondria-targeted ratiometric fluorescent probe, MOBDP-I, for the detection of ONOO^−^. As depicted in Scheme 1, the mitochondria-targeting moiety–indole-salt group was specifically incorporated into the BODIPY scaffold via a carbon–carbon double bond (C=C) to construct the fluorescent probe, MOBDP-I. Upon interaction with ONOO^−^, the C=C bond between the BODIPY backbone and the indole-salt group was oxidized and broken, leading to an 18-fold enhancement of fluorescence at 510 nm, along with a significant fluorescence decrease at 596 nm. The ratio of I_510_/I_596_ was enhanced by approximately 44-fold. More importantly, MOBDP-I was successfully employed to detect ONOO^−^ in inflammatory cells and tissues.

## 2. Experimental Section

### 2.1. Synthesis of MOBDP

4-Hydroxy benzaldehyde (0.54 g, 4.40 mmol), 2, 4-dimethylpyrrole (1.0 mL, 9.74 mmol) and a few drops of TFA were dissolved in THF (75 mL). The reaction mixture was stirred at room temperature overnight under N_2_ atmosphere. DDQ (1.10 g, 4.87 mmol) was then added, and the mixture was stirred for 3 h. Afterward, Et_3_N (7.5 mL) and BF_3_·OEt_2_ (7.5 mL) were added under an ice bath, and the mixture was stirred for an additional 6 h. The solvent was then evaporated, and the residue was extracted with a saturated NaHCO_3_ solution and DCM. The combined organic layers were evaporated under vacuum, and the crude product was purified by column chromatography (PE/EA = 20:1 to 5:1), yielding a red solid (540 mg). Yield: 38%. ^1^H NMR (400 MHz, CDCl_3_): δ 7.17 (d, 2H, *J* = 7.9 Hz), δ 6.98 (d, *J* = 8.1 Hz, 2H), δ 6.01 (s, 2H), δ 2.58 (s, 6H), δ 1.48 (s, 6H). MS (ESI, negative): *m*/*z*. calcd for C_19_H_19_BF_2_N_2_O, 340.16; found 339.2 [M-H]^−^.

### 2.2. Synthesis of MOBDP-CHO

MOBDP-CHO was obtained via the Vilsmeier–Haack reaction. POCl_3_ (3 mL) was slowly added to DMF (3 mL) and stirred at 0 °C for 5 min under N_2_ atmosphere. The mixture was then stirred at room temperature for 30 min. MOBDP (85.3 mg, 0.25 mmol) was dissolved in ClCH_2_CH_2_Cl (50 mL) and added to the mixture, which was stirred at 55 °C for 3 h. After the reaction was complete, a saturated NaHCO_3_ solution (100 mL) was added to adjust the pH to neutral. The reaction mixture was extracted with DCM. The combined organic layers were dried over Na_2_SO_4_ and evaporated under vacuum. The crude product was further purified by column chromatography (DCM/EA = 25:1) to obtain MOBDP-CHO (54.1 mg). Yield: 58.6%.^1^H NMR (400 MHz, DMSO-*d*_6_): δ 9.96 (s, 1H), δ 7.18 (d, 2H, *J* = 8.5 Hz,), δ 6.96 (d, 2H, *J* = 8.5 Hz), δ 6.44 (s, 1H), δ 2.71 (s, 3H), δ 2.54 (s, 3H), δ 1.70 (s, 3H), δ 1.49 (s, 3H). MS (ESI, negative) *m*/*z*. calcd for C_20_H_19_BF_2_N_2_O_2_, 368.1; found 367.1 [M-H]^−^.

### 2.3. Synthesis of MOBDP-I

MOBDP-I was synthesized through a Knoevenagel condensation reaction. MOBDP-CHO (150 mg, 0.41 mmol) and 1,2,3,3-tetramethyl-3H-indole iodide (147 mg, 0.49 mmol) were dissolved in anhydrous ethanol (20 mL). The reaction mixture was refluxed at 85 °C under N_2_ atmosphere for 8 h. After completion, the reaction solution was evaporated under reduced pressure. The crude product was purified by column chromatography (DCM/MeOH = 50:1 to 10:1) to obtain a violet-black solid (120 mg). Yield: 58.6%. ^1^H NMR (400 MHz, DMSO-*d*_6_) δ 10.02 (s, 1H), δ 8.06 (d, 1H, *J* = 16.3 Hz), δ 7.88–7.82 (m, 2H), δ 7.63–7.55 (m, 2H), δ 7.22 (d, *J* = 8.5 Hz, 2H), δ 7.00 (d, 2H, *J* = 8.5 Hz), δ 6.94 (d, 1H, *J* = 16.3 Hz), δ 6.49 (d, 1H, *J* = 1.2 Hz), δ 4.00 (s, 3H), δ 2.82 (s, 3H), δ 2.57 (s, 3H), δ 1.73 (s, 9H), δ 1.53 (s, 3H). ^13^C NMR (101 MHz, DMSO) δ 182.47, 162.19, 159.18, 157.35, 155.55, 148.02, 144.87, 144.05, 143.43, 142.33, 140.52, 134.53, 134.51, 131.43, 129.75, 129.41, 129.24, 125.69, 125.62, 125.06, 123.96, 123.30, 116.77, 115.16, 111.51, 55.40, 52.89, 40.63, 40.42, 40.21, 40.01, 39.80, 39.59, 39.38, 34.45, 26.85, 15.31, 14.28, 13.62. HRMS (ESI, positive) *m*/*z*. calcd for C_32_H_33_BF_2_N_3_O^+^ ([M]^+^): 524.2679; found: 524.2680.

## 3. Result and Discussion

### 3.1. Design and Synthesis of Fluorescent Probe

BODIPY-based fluorescent probes have been developed for the detection of ONOO^−^ through structural modifications of the BODIPY core [26,27,28,29]. ONOO^−^ exhibits strong oxidizing properties, enabling the oxidation and cleavage of unsaturated bonds. This mechanism has been widely employed in the design of fluorescent probes for ONOO^−^ sensing [30,31,32,33]. The negatively charged mitochondrial inner membrane can be targeted by the positively charged Indo group, which also exhibits strong electron-withdrawing properties. When the Indo group is conjugated to the BODIPY fluorophore via a C=C bond, the emission wavelength of the fluorescent probe is red-shifted. In this study, a BODIPY-based, mitochondria-targeting, ratiometric fluorescent probe was synthesized according to the steps outlined in Figure S1. The detailed synthesis is described in Experimental Section. The corresponding ^1^H NMR, ^13^C NMR and HRMS characterizations are provided in Figures S2–S8.

### 3.2. Photophysical Properties and Responses to ONOO^−^

**Figure 1:**
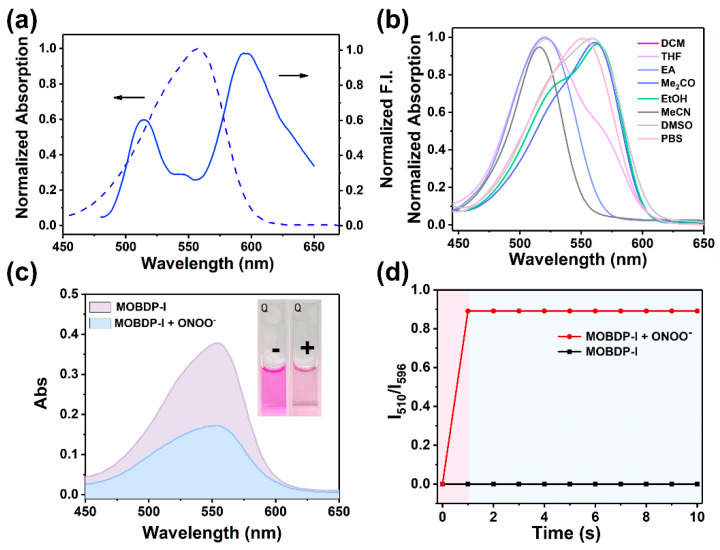
(**a**) UV-vis absorption and fluorescence spectra of MOBDP-I (10 μM) in PBS (10 mM, pH = 7.4, 0.1% DMSO). (**b**) Normalized absorption spectra of MOBDP-I (10 μM) in various solvents, including DCM, THF, EA, Me_2_CO, EtOH, MeCN, DMSO, PBS. (**c**) Absorption spectra of MOBDP-I (10 μM) with and without ONOO^−^ (10 μM) in PBS (pH = 7.4). Inset: − (without ONOO^−^), + (with ONOO^−^). (**d**) The response time of MOBDP-I (10 μM) to ONOO^−^ (10 μM) in PBS (pH 7.4), λ_ex_ = 460 nm.

**Figure 2:**
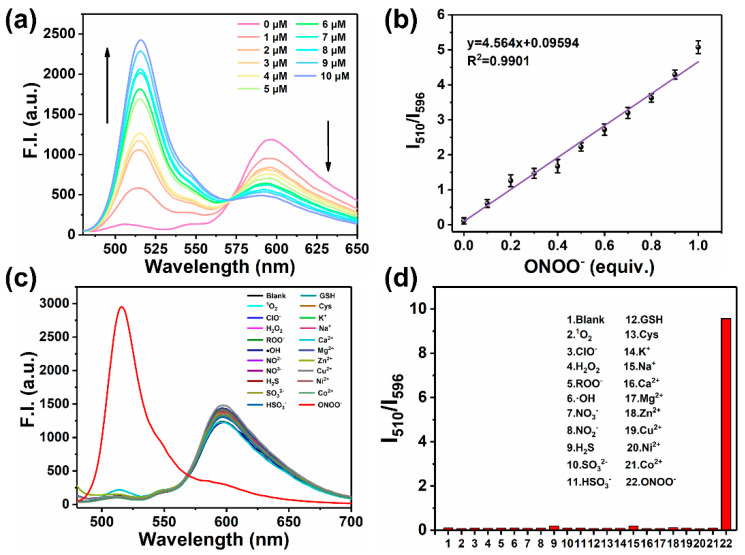
(**a**) Fluorescence spectra of MOBDP-I (10 μM) in response to ONOO^−^ (0~10 μΜ) in PBS (pH = 7.4), λ_ex_ = 460 nm. (**b**) Linear relationship between the fluorescence intensity ratio (I_510_/I_596_) and ONOO^−^ concentration from 0 to 10 μM. (**c**) Fluorescence spectra of MOBDP-I (10 μM) in the presence of 10 μM of ONOO^−^ or 50 μM of other relevant analytes. (**d**) The fluorescence response intensity (I_510_/I_596_) of MOBDP-I to ONOO^−^ (10 μM) and other relevant analytes (50 μM). Analytes: ^1^O_2_, ClO^−^, H_2_O_2_, ROO^−^, ·OH, NO_3_^−^, NO_2_^−^, H_2_S, SO_3_^2−^, HSO_3_^−^, GSH, Cys, K^+^, Na^+^, Ca^2+^, Mg^2+^, Zn^2+^, Cu^2+^, Ni^2+^, Co^2+^. Samples were measured in PBS (pH = 7.4) at room temperature with λ_ex_ = 460 nm. The error bars represent ± standard deviation (SD) (n = 3).

After obtaining MOBDP-I, we began exploring its spectral properties in PBS buffer (pH 7.4, 10 mM). We first examined the absorption and fluorescence emission spectra of MOBDP-I. As shown in Figure 1a, MOBDP-I exhibited a maximum absorption peak at 550 nm and two major emission peaks at 510 nm and 596 nm. We tested the absorption spectra of MOBDP-I in various solvents at room temperature (Figure 1b). The absorption maxima of MOBDP-I in highly polar solvents such as PBS, DMSO, and methanol exhibited a red-shift relative to those in less polar solvents like DCM. MOBDP-I is a molecule with a D-π-A structure. The red-shift of MOBDP-I absorption spectra could be induced by solvent polarity, indicating that the ICT effect exists in the structure of MOBDP-I. The reaction of MOBDP-I to ONOO^−^ was investigated at pH 7.4 in aqueous solutions. In the presence of ONOO^−^, the absorption intensity at 550 nm decreased, and the solution color changed from purple to light pink (inset in Figure 1c), due to the oxidation of the double bond by ONOO^−^ (Figure 1c). MOBDP-I responded to ONOO^−^ rapidly, and the reaction completed in less than 1 s, indicating that MOBDP-I could quickly and effectively detect ONOO^−^ (Figure 1d). As shown in Figure 2a, with increasing concentrations of ONOO^−^, the fluorescence intensity at 596 nm decreased, while the fluorescence intensity at 510 nm gradually increased. This indicated that MOBDP-I was capable of detecting ONOO^−^ and exhibited a ratiometric fluorescence signal. The change in ratiometric fluorescence of MOBDP-I was linearly related to the concentration of ONOO^−^ in the range of 0 to 10 μM (Figure 2b). The correct linear equation was y = 4.564x + 0.09594, with a linear regression coefficient (R^2^) of 0.9901, indicating a strong linear relationship. Based on the formula LOD = 3σ/s, the limit of detection (LOD) of MOBDP-I for ONOO^−^ was determined to be 9.6 nM, confirming that MOBDP-I could sensitively detect ONOO^−^.

Considering the complexity of the physiological environment and the potential impact on sensing signals, we evaluated the selectivity of MOBDP-I for various potential coexisting molecules, including reducing agents (H_2_S, SO_3_^2−^, HSO_3_^−^, GSH, Cys), oxidants (^1^O_2_, ClO^−^, H_2_O_2_, ROO^−^, ·OH, NO_2_^−^, NO_3_^−^), and metal ions (K^+^, Na^+^, Ca^2+^, Mg^2+^, Zn^2+^, Cu^2+^, Ni^2+^, Co^2+^). As shown in Figure 2c,d, these interfering species exhibited negligible fluorescence changes compared to ONOO^−^. Furthermore, MOBDP-I demonstrated stable ratiometric fluorescence enhancement for ONOO^−^ even in the presence of high concentrations of interferents (Figure S9). These results confirm that MOBDP-I exhibits excellent selectivity for ONOO^−^. In addition, the key analytical parameters of MOBDP-I (including maximum λ_ex_/λ_em_ of fluorophore, signal-to-noise ratio (SNR), detection limit, response time, and biological applications) were compared with previously reported fluorescent probes for detecting ONOO^−^ (Table S1). The comprehensive data analysis revealed that the probe MOBDP-I exhibited superior analytical performance in terms of SNR, detection limit, response time, and biological applications. To investigate the mechanism of the reaction between MOBDP-I and ONOO^−^, we characterized the reaction product using ESI-MS (Figure S10). The molecular weight of the product (*m*/*z* = 367.3, [M-H]^−^) was found to be consistent with that of MOBDP-CHO. As shown in Figures S11 and S12 the emission spectrum of MOBDP-CHO matches that of the product formed in the reaction between MOBDP-I and ONOO^−^, with the maximum emission wavelength at 510 nm. These results confirmed that ONOO^−^ oxidatively cleaved the C=C bond of MOBDP-I, releasing the intermediate product MOBDP-CHO.

### 3.3. Mitochondrial Localization

**Figure 3:**
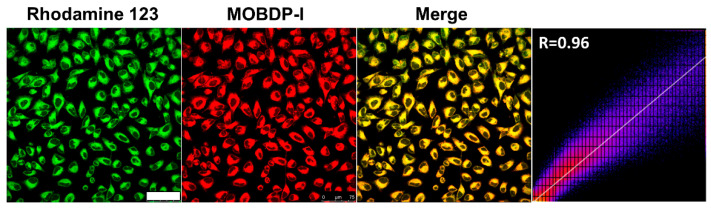
Co-localization imaging in HeLa cells with probe MOBDP-I (10 μM) and Rhodamine 123 (5 μM). Green channel: λ_ex_ = 488, λ_em_ = 510–540 nm. Red channel: λ_ex_ = 543, λ_em_ = 580–650 nm, scale bar: 75 μm.

The cytotoxicity of MOBDP-I on HeLa, RAW264.7, and PC12 cells was assessed using methyl thiazolyl tetrazolium (MTT) assay prior to cell imaging [34]. The cells survival rates remained above 80% even at a MOBDP-I concentration of 40 μM, indicating that MOBDP-I presented low cytotoxicity (Figure S13). The photostability of MOBDP-I is shown in Figure S14. After 300 s of continuous laser irradiation, the average fluorescence intensity of MOBDP-I decreased by only 24%, demonstrating good photostability and suitability for both cellular and in vivo imaging. Given that the structure of MOBDP-I contains positively charged indole-salt moieties, MOBDP-I possessed mitochondrial localization capability. Co-localization experiments were performed with the commercial mitochondrial dye Rhodamine 123 (Rh123) as a mitochondrial tracker. The mitochondrial tracker has an excitation wavelength of 488 nm and a fluorescence collection range of 510 to 540 nm. Fluorescence confocal imaging revealed a high Pearson correlation coefficient (R = 0.96, calculated by ImageJ (Version: Fiji) software) between MOBDP-I and the mitochondrial tracker (Figure 3), indicating that MOBDP-I could effectively target mitochondria.

### 3.4. Cell Imaging of Exogenous and Endogenous ONOO^−^

**Figure 4:**
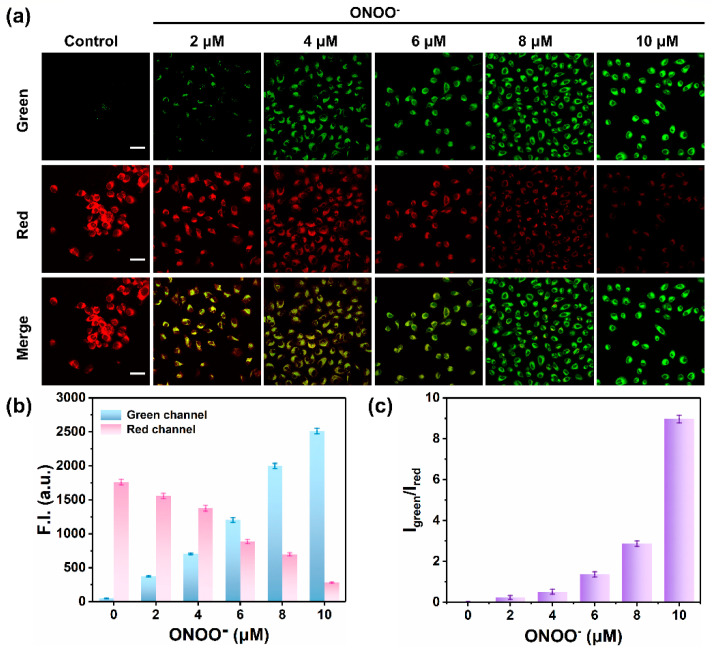
(**a**) Fluorescence imaging of exogenous ONOO^−^ (0, 2, 4, 6, 8, and 10 μM) in HeLa cells stained with MOBDP-I (10 μM). scale bar: 50 μm. (**b**) Relative fluorescence intensities corresponding to panel (**a**) in both the green and red channels. (**c**) Fluorescence intensity ratio corresponding to panel (**a**). Green channel: λ_ex_ = 488 nm, λ_em_ = 510–540 nm. Red channel: λ_ex_ = 543 nm, λ_em_ = 580–650 nm. The error bars represent ± standard deviation (SD) (n = 3).

Encouraged by the excellent properties of MOBDP-I for detecting ONOO^−^ in solutions, we first investigated its ability to detect exogenous ONOO^−^ in living cells. As shown in Figure 4a,b, when HeLa cells were incubated with only the probe MOBDP-I, the red channel exhibited obvious fluorescence, while the green channel displayed almost no fluorescence. As the concentration of ONOO^−^ increased, the fluorescence intensity of the green channel progressively increased, while that of the red channel gradually decreased. At a concentration of 10 μM ONOO^−^, the fluorescence intensity of the green channel was enhanced by more than 49-fold, while the fluorescence intensity of the red channel decreased by 84%. Therefore, there was a more than 310-fold enhancement in the ratiometric fluorescence intensity (Figure 4c). MOBDP-I was effective for monitoring exogenous ONOO^−^ levels in living cells.

**Figure 5:**
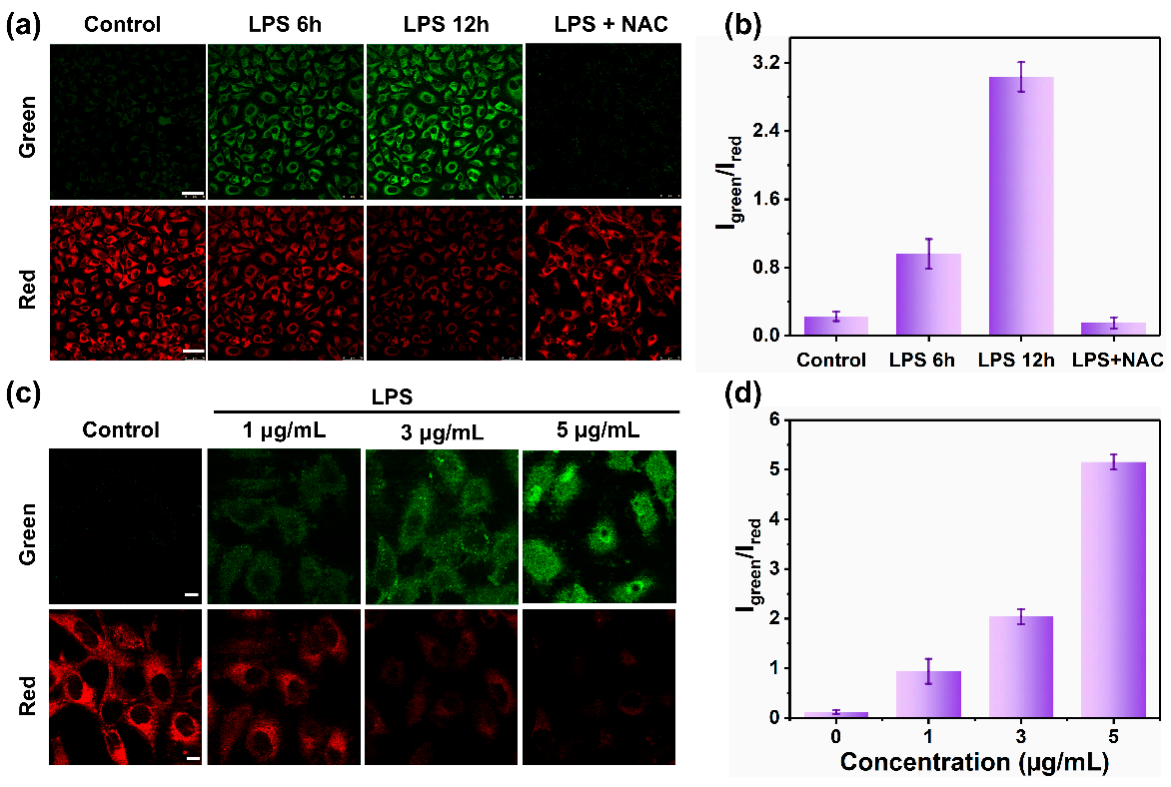
Fluorescence imaging of endogenous ONOO^−^ in HeLa cells. (**a**) The cells were treated with LPS (1 μg/mL) for different times and then incubated with MOBDP-I (10 μM). Green channel: λ_ex_ = 488 nm, λ_em_ = 510–540 nm. Red channel: λ_ex_ = 543 nm, λ_em_ = 580–650 nm, scale bar: 50 μm. (**b**) Fluorescence intensity ratio corresponding to panel (**a**). (**c**) The cells were treated with different concentrations of LPS for 6 h and then incubated with MOBDP-I (10 μM). Green channel: λ_ex_ = 488 nm, λ_em_ = 510–540 nm. Red channel: λ_ex_ = 543 nm, λ_em_ = 580–650 nm, scale bar: 10 μm; (**d**) Fluorescence intensity ratio corresponding to panel (**c**). The error bars represent ± standard deviation (SD) (n = 3).

We further investigated the ability of MOBDP-I to detect endogenous ONOO^−^ in cells. HeLa cells were stimulated with lipopolysaccharide (LPS) (1 μg/mL) to induce the production of endogenous ONOO^−^. As shown in Figure 5a, control cells exhibited weak fluorescence in the green channel and strong fluorescence in the red channel after co-incubation with MOBDP-I. As the co-incubation time with LPS increased from 6 to 12 h, the fluorescence intensity of the green channel gradually increased, while that of the red channel decreased. Compared to the control group, the ratiometric fluorescence intensity of the treatment group (LPS 12 h) increased approximately 12-fold. N-acetylcysteine (NAC) effectively removed ONOO^−^ from the cells, resulting in fluorescence intensities similar to those of the control group (Figure 5b). Similar imaging results were observed in RAW264.7 cells (Figure S15). As shown in Figure 5c, the red channel fluorescence intensity gradually decreased, while the green channel fluorescence intensity increased with increasing LPS concentrations. Quantitative analysis clearly demonstrated significant changes in the ratiometric signal of the dual-channel fluorescence intensities (Figure 5d). These results suggested that MOBDP-I can effectively monitor the levels of both endogenous and exogenous ONOO^−^ in living cells.

### 3.5. Fluorescent Imaging of ONOO^−^ in Inflamed Mouse Model

**Figure 6:**
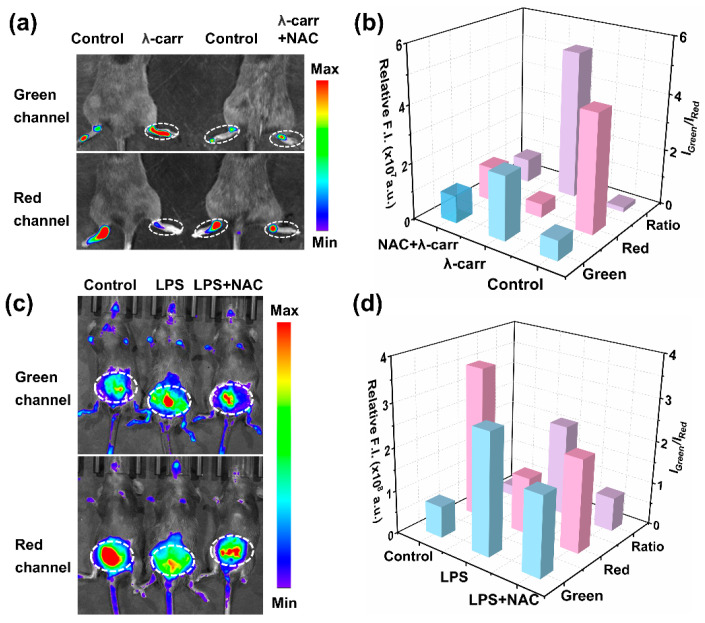
(**a**) Fluorescence imaging of MOBDP-I in a mouse model of rheumatoid arthritis. Control group: the left hind limb of mice, in situ injection of PBS (50 μL, 10 mM, pH = 7.0); experimental group: the right hind limb of mice, in situ injection of λ-carr (50 μL, 5 mg/mL) or λ-carr (50 μL, 5 mg/mL) + NAC (50 μL, 30 mg/kg). (**b**) Relative fluorescence intensities corresponding to panel (**a**); (**c**) Fluorescence imaging of MOBDP-I in a mouse model of peritonitis. Control group: intraperitoneal injection of PBS (50 μL, 10 mM, pH = 7.0); experimental group: intraperitoneal injection of LPS (50 μL, 2 mg/mL) or LPS (50 μL, 2 mg/mL) + NAC (50 μL, 30 mg/kg). (**d**) Relative fluorescence intensities corresponding to panel (**c**). Green channel, λ_ex_ = 460 nm, λ_em_ = 510–550 nm. Red channel, λ_ex_ = 570 nm, λ_em_ = 650–700 nm.

Building on the exceptional ability of MOBDP-I to image intracellular ONOO^−^, we further investigated its potential for in vivo imaging. Various inflammatory mouse models were applied to evaluate sensing capability. First, we assessed the imaging performance of MOBDP-I using an arthritis model. We used λ-carrageenan (λ-carr) to induce a mouse model of arthritis [35,36,37,38,39]. To construct the arthritis model, C57BL/6 mice were injected with λ-carr (50 μL, 5 mg/mL) in the right hindlimb joint. PBS (50 μL) was injected into the left hindlimb joint as a control group. After 8 h, the right hind limb of the mice appeared red and swollen, whereas the control group did not show this phenomenon, indicating that the arthritis model was successfully constructed. MOBDP-I (20 μM, 100 μL in 1:9 DMSO/PBS *v*/*v*) was injected and imaged with a three-dimensional optical in vivo imaging system (IVIS Spectrum). As shown in Figure 6a, in the control group, the red channel at the joint displayed clear fluorescence, while the green channel exhibited almost no signal. In contrast, the experimental group showed a reduction in the red channel fluorescence intensity, whereas the green channel intensity was significantly enhanced, resulting in a six-fold increase in the ratio of fluorescence intensities in the two channels (Figure 6b). To confirm that this phenomenon was due to ONOO^−^ production, NAC was administered to the experimental group. The fluorescence of the red channel was restored, while the green channel fluorescence was weakened, confirming the effect of ONOO^−^. To further investigate the broader applicability of MOBDP-I for ONOO^−^ imaging in inflamed tissues, we established a mouse peritonitis model. As shown in Figure 6c,d, the fluorescence intensity in the green channel of LPS-injected mice was significantly higher than that of the control group, while the red channel showed a lower intensity compared to the control. This suggests that peritonitis induces an increase in ONOO^−^. Following NAC treatment, the fluorescence intensity in the green channel markedly decreased, whereas the red channel intensity significantly increased, indicating that NAC effectively eliminated ONOO^−^. T MOBDP-I could sense the change in ONOO^−^ in the mouse peritonitis model.

**Figure 7:**
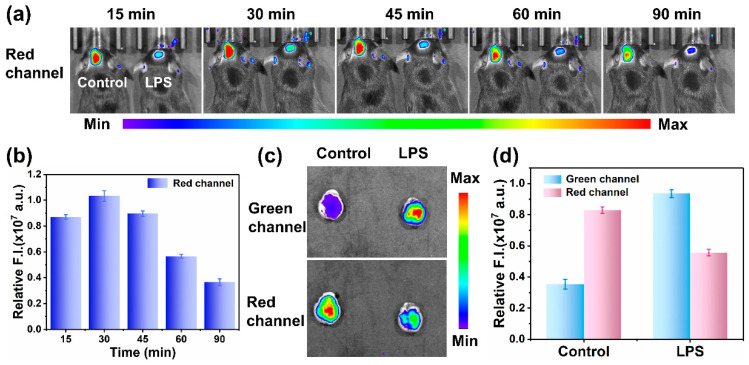
(**a**) Fluorescence imaging of MOBDP-I in mouse model of brain inflammation. Control group (left): tail vein injection of PBS (50 μL, 10 mM, pH = 7.0), and then MOBDP-I (20 μM, 100 μL in 1:9 DMSO/PBS *v*/*v*); experimental group (right): tail vein injection of LPS (100 μL, 2 mg/mL, 8 h), and then MOBDP-I (20 μM, 100 μL in 1:9 DMSO/PBS *v*/*v*). (**b**) Relative fluorescence intensities corresponding to the LPS-treated group in panel (**a**). Red channel, λ_ex_ = 570 nm, λ_em_ = 650–700 nm. (**c**) Fluorescence imaging of the dissected mouse brains. (**d**) Relative fluorescence intensities corresponding to panel (**c**). Green channel, λ_ex_ = 460 nm, λ_em_ =510–550 nm. Red channel, λ_ex_ = 570 nm, λ_em_ = 650–700 nm. The error bars represent ± standard deviation (SD) (n = 3).

Moreover, we were curious whether the MOBDP-I probe can be used to track the levels of ONOO^−^ in brain inflammation. Therefore, we constructed a mouse brain inflammation model. Brain inflammation could be induced by intraperitoneal injection of LPS (2 mg/mL,100 μL) [40,41,42,43]. MOBDP-I was used to image changes in ONOO^−^ in brain inflammation. As shown in Figure 7a,b, the fluorescence intensity in the red channel of the experimental group (right) was significantly stronger than that of the control group (left), indicating that MOBDP-I successfully crossed the blood–brain barrier and imaged ONOO^−^ in brain-inflammation mice. During the 90 min imaging session, the fluorescence intensity initially increased and then gradually decreased. The reason for this was that the brain uptake rate of MOBDP-I exceeded its clearance rate during the first 30 min, leading to the accumulation of the probe in the brain with enhanced fluorescence. Subsequently, the fluorescence intensity gradually decreased because of brain clearance and metabolic mechanisms. However, imaging results in the green channel were not observable. The reason for this was that emission wavelength of the MOBDP-I green channel was too short to pass through the structure of the mouse skull. When mouse brains were dissected for in vitro fluorescence imaging, the signal of MOBDP-I from both green and red channels could be detected in the dissected mouse brains, with a significant increase in the fluorescence intensity of the green channel and a decrease in the red channel (Figure 7c,d). The biosafety of the probe MOBDP-I was evaluated. The hemolysis rate of MOBDP-I was relatively low (Figure S16). The metabolic distribution of MOBDP-I in various organs was examined, and histopathological analyses of the major organs were performed using H&E staining. MOBDP-I was primarily metabolized in the liver and had no significant effects on other organs (Figures S17 and S18). These results suggest that MOBDP-I possessed a favorable biosafety profile. MOBDP-I could detect ONOO^−^ sensitively and has feasibility at the organismal level, and holds strong potential as a candidate for future clinical studies.

## 4. Conclusions

In summary, we have developed a ratiometric sensing platform that selectively targets ONOO^−^ in mitochondria. In vitro experiments demonstrated that MOBDP-I exhibited a significant spectral response to ONOO^−^. In the presence of ONOO^−^, the probe showed an enhancement of fluorescence at 510 nm, along with a fluorescence decrease at 596 nm, enabling the quantification of ONOO^−^ levels by the ratio of fluorescence intensities from the two channels. MOBDP-I has the advantages of high anti-interference, low cytotoxicity, high sensitivity, and rapid response. Moreover, MOBDP-I can be applied for the ratiometric monitoring of mitochondrial ONOO^−^ and assessing the mitochondrial oxidative stress status in living cells. Furthermore, in vivo experiments indicated that MOBDP-I can effectively distinguish between inflammatory and normal tissues in mouse models of rheumatoid arthritis, peritonitis, and brain inflammation. We believe MOBDP-I holds significant potential to be an effective molecular tool to study the relationship between ONOO^−^ and the occurrence and development of inflammatory diseases.

## Data Availability

The data presented in this study are available on request from the corresponding author.

## Supplementary Materials

The following supporting information can be downloaded at: https://www.mdpi.com/article/10.3390/bios14120638/s1. Figure S1. The synthetic route of MOBDP-I. Figure S2. ^1^H NMR spectrum of MOBDP in CDCl_3_-*d*_4_. Figure S3. ^1^H NMR spectrum of MOBDP-CHO in CDCl_3_-*d*_4_. Figure S4. ^1^H NMR spectrum of MOBDP-I in DMSO-*d*_6_. Figure S5. ^13^C NMR spectrum of MOBDP-I in DMSO-*d*_6_. Figure S6. The mass spectrum (ESI) of MOBDP. Figure S7. The mass spectrum (ESI) of MOBDP-CHO. Figure S8. The mass spectrum (HRMS) of MOBDP-I. HRMS (ESI, positive) *m*/*z*. calcd for C_32_H_33_BF_2_N_3_O^+^ ([M]^+^): 524.2679; found: 524.2680. Figure S9. The fluorescence response intensity (I_510_/I_596_) of MOBDP-I to various analytes (50 μM) and ONOO^−^ (10 μM) + analytes (50 μM). Analytes (1–20): ^1^O_2_, ClO^-^, H_2_O_2_, ROO^−^, ·OH, NO_3_^−^, NO_2_^−^, H_2_S, SO_3_^2−^, HSO_3_^−^, GSH, Cys, K^+^, Na^+^, Ca^2+^, Mg^2+^, Zn^2+^, Cu^2+^, Ni^2+^, Co^2+^. Figure S10. The reaction mechanism of MOBDP-I with ONOO^−^ and mass spectra (ESI) of MOBDP-I and MOBDP-CHO. Note that MOBDP-I was analyzed in positive ion mode. The detected charged species was [M]^+^, as itself is a cation. The product MOBDP-CHO was analyzed in negative ion mode. The detected charged species should be [M-H]^−^. Figure S11. Fluorescence spectra of MOBDP-CHO (10 μM), MOBDP-I (10 μM) and MOBDP-I (10 μM) in response to ONOO^−^ (10 μΜ) in PBS (pH = 7.4). Figure S12. Fluorescence spectra of MOBDP-I (10 μM), MOBDP-I (10 μM) + H_2_O_2_ (50 μΜ), MOBDP-I (10 μM) + ONOO^−^ (10 μΜ) and MOBDP-I (10 μM) + H_2_O_2_ (50 μΜ) + ONOO^−^ (10 μΜ). Figure S13. MTT assay of (a) HeLa cells, (b) RAW264.7 cells and (c) PC12 cells in the presence of different concentrations of MOBDP-I (0~40 μM). The error bars represent ± standard deviation (SD) (n = 3). Figure S14. (a) Photostability of MOBDP-I (10 μM) and Rhodamine 123 (5 μM) incubated with HeLa cells, the signal intensity varies with the laser bleaching time. (Red channel: λ_ex_ =543 nm, λ_em_ = 580–650 nm. Green channel: λ_ex_ = 488 nm, λ_em_ = 510–540 nm, scale bar = 50 μm). (b) Normalized intensity profiles of average intracellular signals calculated from the confocal fluorescence images in (a) by applying Image*J*. Figure S15. Confocal fluorescence imaging of ONOO^−^ in RAW264.7 cells using the probe MOBDP-I (10 μM, 30 min). Control group: MOBDP-I only; LPS group: LPS (1 μg/mL, 6 h) then MOBDP-I; LPS + Minocycline group: LPS (1 μg/mL, 6 h), minocycline (300 μM, 2 h), then MOBDP-I; ONOO^−^ group: exogenous ONOO^−^ (10 μM, 30 min), then MOBDP-I. Green channel: λ_ex_ = 488 nm, λ_em_ = 510–540 nm. Red channel: λ_ex_ = 543 nm, λ_em_ = 600–650 nm, scale bar: 75 μm. Figure S16. Hemolysis rate at different concentrations of MOBDP-I (0–40 μM). Figure S17. Fluorescence imaging in the major organs (heart, liver, spleen, lung, and kidney) isolated from mice. Figure S18. Hematoxylin and eosin (H&E) staining of major organs (heart, liver, spleen, lung, and kidney) collected from mice. Table S1. Comparison of the proposed probe with other reported fluorescence probes for detecting peroxynitrite. References [15,21,43,44,45,46,47,48,49] are cited in Supplementary Materials.
