# Supplementary material for: BODIPY-Based Ratiometric Fluorescent Probe for Sensing Peroxynitrite in Inflammatory Cells and Tissues
Source: Biosensors (Basel). 2024 Dec 22;14(12):638. doi: 10.3390/bios14120638 (PMC11674116; doi:10.3390/bios14120638)
Supplement: Supplementary file 1 [file biosensors-14-00638-s001.zip › biosensors-3316498-supplementary.pdf]

Supporting Information

BODIPY-Based Ratiometric Fluorescent Probe for Sensing Peroxynitrite in Inflammatory Cells and Tissues

Qian Wu, Ziwei Hu, Guoyang Zhang, Yulong Jin * and Zhuo Wang *

State Key Laboratory of Chemical Resource Engineering, College of Chemistry, Beijing University of Chemical Technology, Beijing 100029, China

* Correspondence: jinyulong@buct.edu.cn (Y.J.); wangzhuo77@mail.buct.edu.cn (Z.W.)

Reagent and Materials

All reagents were commercially obtained and used without further purification unless otherwise specified. Deionized water was used for all experiments. 4-hydroxybenzaldehyde, 2,4-dimethylpyrrole, trifluoroacetic acid (TFA), 2,3-dichloro-5,6-dicyano-1,4-benzoquinone (DDQ), triethylamine (Et_3N), boron trifluoride diethyl etherate ($BF_3 \cdot OEt_2$), N,N-dimethylformamide (DMF), phosphorus oxychloride ($POCl_3$), 1,2,3,3-tetramethyl-3H-indolium iodide (Indo), Rhodamine 123 (Rh 123), lipopolysaccharide (LPS), carbonyl cyanide-3-chlorophenylhydrazone (CCCP), λ-carrageenan (λ-carr), dimethyl sulfoxide (DMSO), hydrogen peroxide (H_2O_2), hydrochloric acid (HCl), sodium nitrite ($NaNO_2$), and sodium hydroxide (NaOH) were purchased from Beijing Innochem Technology Co., Ltd. Thin-layer chromatography (TLC) plates and silica gel were supplied by Qingdao Ocean Chemical.

All cell culture reagents were sterile and handled in a biosafety cabinet to ensure a contamination-free environment. High-glucose Dulbecco's Modified Eagle's Medium (DMEM), heat-inactivated fetal bovine serum (FBS), penicillin-streptomycin, and phosphate-buffered saline (PBS) were purchased from Hyclone. The human blood sample was provided by Beijing China-Japan Friendship Hospital. The 3-(4,5-dimethylthiazol-2-yl)-2,5-diphenyltetrazolium bromide (MTT) kit was obtained from KeyGEN BioTECH (Jiangsu, China).

Instruments

^1H NMR and ^{13}C NMR spectra were recorded on a Bruker Avance III spectrometer (400 MHz, Germany). UV−vis spectra were recorded using a UV−vis spectrophotometer (HITACHI, U3900H, Japan), and fluorescence spectra were obtained on a fluorescence spectrophotometer (HITACHI, F-4600, Japan). A laser scanning confocal microscope (Leica SP8, Germany) was used for cell imaging. Cell viability measurements were recorded using a microplate reader (PerkinElmer Enspire, USA). Fluorescence signals from the mice were captured using an IVIS Lumina IV system (PerkinElmer Enspire, USA).

Sample Preparation and Testing.

The probe MOBDP-I was dissolved in DMSO to prepare a stock solution (5 mM). The stock solution of $ONOO^-$ was prepared by simultaneously adding 0.6 M $NaNO_2$, 0.6 M HCl, and 0.7 M H_2O_2 to a 1.5 M NaOH solution at 0 °C. The concentration of $ONOO^-$ was determined by measuring its absorbance at 302 nm, using an extinction coefficient of 1670 $M^{-1} \cdot cm^{-1}$. Fluorescence and absorption measurements were conducted in PBS (10 mM, pH 7.4) with a final concentration of 10 μM for MOBDP-I.

Cell Culture

HeLa cells and RAW264.7 cells were purchased from the Chinese National Infrastructure of Cell Line Resource. HeLa cells and RAW264.7 cells were cultured in high-glucose DMEM supplemented with 10% FBS and 1% antibiotics (penicillin and streptomycin). The cells were incubated at 37 °C in a 5% CO_2 environment. Before cell staining, all cells were plated in a confocal petri dish and cultured for 24 h.

Cytotoxicity Assays

The effects of MOBDP-I on cell viability were assessed using the standard MTT assay. HeLa cells and RAW264.7 cells in the logarithmic growth phase were seeded into 96-well plates at a density of approximately 1×10^4 cells per well. After 24 h of cell attachment, the original medium was removed, and the cells were washed with PBS buffer (pH 7.4). 100 μL of MOBDP-I (0 - 40 μM) was added to each well and incubated for 24 h. Subsequently, 50 μL of MTT solution was

added to each well. After 4 h of incubation, the culture medium was removed, and 150 μL of DMSO was added to dissolve the resulting formazan crystals. After 10 min, absorbance was measured at 490 nm using a microplate reader. Cell viability (%) was calculated using the formula:

$$\text{Cell viability (\%)} = \frac{OD_{sample}-OD_{blank}}{OD_{control}-OD_{blank}} \times 100 \tag{S1}$$

OD $_{sample}$, OD $_{control}$, and OD $_{blank}$ denote the cells incubated with various concentrations of the MOBDP-I, the cells without the MOBDP-I, and the wells containing only the culture media, respectively.

Hemolysis Experiment

Blood samples from mice were collected using vacuum blood collection tubes. 2 mL PBS (pH = 7.4) was added to 1 mL blood sample, centrifuged at 8000 rpm for 10 min, and red blood cells in serum were separated. Then the red blood cells were washed with PBS for 5 times and resuspended in 10 mL PBS. 0.2 mL of the hemoglobin dispersion was mixed with (a) 0.8 mL of PBS as a negative control, (b) 0.8 mL of deionized water as a positive control, and (c) 0.8 mL of different concentrations of probe MOBDP-I. After thorough mixing, the samples were allowed to stand at room temperature for 4 h. The samples were then centrifuged at 12000 rpm for 5 min, and hemolysis was assessed by measuring the UV absorbance of the supernatant at 540 nm using a microplate reader. The hemolysis rate was calculated using the following formula:

$$HR = \frac{Abs_{sample}-Abs_{PBS}}{Abs_{H_2O}-Abs_{PBS}} \times 100\% \tag{S2}$$

Cell Imaging Experiment

In cell imaging experiments of exogenous $ONOO^-$, HeLa cells (3×10^5) were inoculated into confocal culture dishes and incubated for 24 h. The experiment was divided into six groups. The first group of HeLa cells was incubated with MOBDP-I (10 μM) for 30 min. The remaining five groups of cells were first incubated with MOBDP-I (10 μM) for 30 min, and then co-incubated with different concentrations of $ONOO^-$ (2, 4, 6, 8 and 10 μM) for 30 min. Confocal fluorescence imaging of HeLa cells was measured green and red fluorescence channel.

In the cell imaging experiments of endogenous $ONOO^-$, HeLa cells or RAW264.7 cells (3×10^5) were inoculated into confocal culture dishes and incubated for 24 h. The experiment was divided into four groups. In the first group, cells were incubated with MOBDP-I (10 μM) for 30 min. In the second group, cells were pretreated with LPS (3 μg/mL) for 6 h, followed by incubation with MOBDP-I (10 μM) for 30 min. The third group was pretreated with LPS (3 μg/mL) for 12 h, then incubated with MOBDP-I (10 μM) for 30 min. In the fourth group, cells were pretreated with LPS (3 μg/mL) for 6 h, treated with NAC (200 μM) for 2 h, and finally incubated with MOBDP-I (10 μM) for 30 min. Subsequently, the effects of different concentrations of LPS on cells were explored. HeLa cells were incubated with different concentrations of LPS (0, 1, 3 and 5 μg/mL) for 3 h, and then co-incubated with MOBDP-I for 30 min. HeLa cells without LPS were used as the control group, and HeLa cells with different concentrations of LPS were used as the experimental group.

Fluorescence Imaging *in vivo*

C57BL/6 mice (6 - 7 weeks, black, female) were used for fluorescence imaging *in vivo*. The mice were purchased from Beijing Huafukang Biotechnology Co., Ltd. ll animal protocols were approved by the Animal Care Committee of China-Japan Friendship Hospital, with approval number zryhyy12-20-10-2. According to the relevant literature, a certain concentration of λ-

Carrageenan (λ-carr) can produce $ONOO^-$ at the joints of mice and induce rheumatoid arthritis[1]. while a certain concentration of LPS can generate $ONOO^-$ in the abdominal area, leading to peritonitis[2]. Additionally, LPS at specific concentrations can cause $ONOO^-$ production in the brain of mice, inducing an inflammatory response.[3] Before performing *in vivo* fluorescence imaging, the hair at the imaging site was removed to minimize interference with the imaging results. For the mouse rheumatoid arthritis and peritonitis models, MOBDP-I was injected directly at the site of inflammation, while for the brain inflammation model, MOBDP-I was administered intravenously. The imaging process utilized 5% isoflurane gas with an oxygen flow rate of 1.0 L/min. Fluorescence intensity from the imaging area was analyzed using Living Image software (Life Software 4.5.5).

Imaging of Arthritis: the mice were divided into two groups. The left limb of each mouse was used as a control, with an in-situ injection of PBS (50 µL, pH = 7). The right limb was treated with an in-situ injection of λ-carr (50 µL, 5 mg/mL) in the right hind limb joint to induce arthritis. Eight hours later, one mouse received an in-situ injection of NAC (50 µL, 30 mg/kg) into the right hind limb joint. One hour after that, all mice received an in-situ injection of MOBDP-I (20 µM, 50 µL in 1:9 DMSO/PBS) into the hind limb joints, followed by anesthesia for in vivo fluorescence imaging.

Imaging of Peritonitis: The mice were divided into three groups. The control group received an intraperitoneal injection of PBS (50 µL, pH 7.4). Experimental groups A and B were injected intraperitoneally with LPS (50 µL, 2 mg/mL) to induce peritonitis. Eight hours later, mice in experimental group B were administered an intraperitoneal injection of NAC (50 µL, 30 mg/mL). One hour after NAC administration, all mice received an intraperitoneal injection of MOBDP-I (20 µM, 50 µL in 1:9 DMSO/PBS). The abdominal region of each mouse was then imaged using a 3D quantitative imaging system.

Imaging of Brain Inflammation: The mice were randomly assigned to two groups. The control group received an intraperitoneal injection of PBS (100 µL, pH 7), while the experimental group was injected intraperitoneally with LPS (100 µL, 2 mg/mL) to induce brain inflammation. After 8 hours, all mice were injected with MOBDP-I (20 µM, 100 µL of 1:9 DMSO/PBS v/v) via the tail vein. Subsequently, the mice were euthanized, and their brains were harvested for *in vitro* fluorescence imaging.

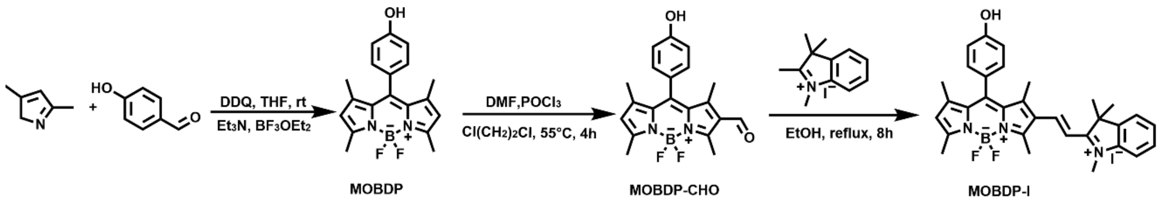

Figure S1. The synthetic route of MOBDP-I.

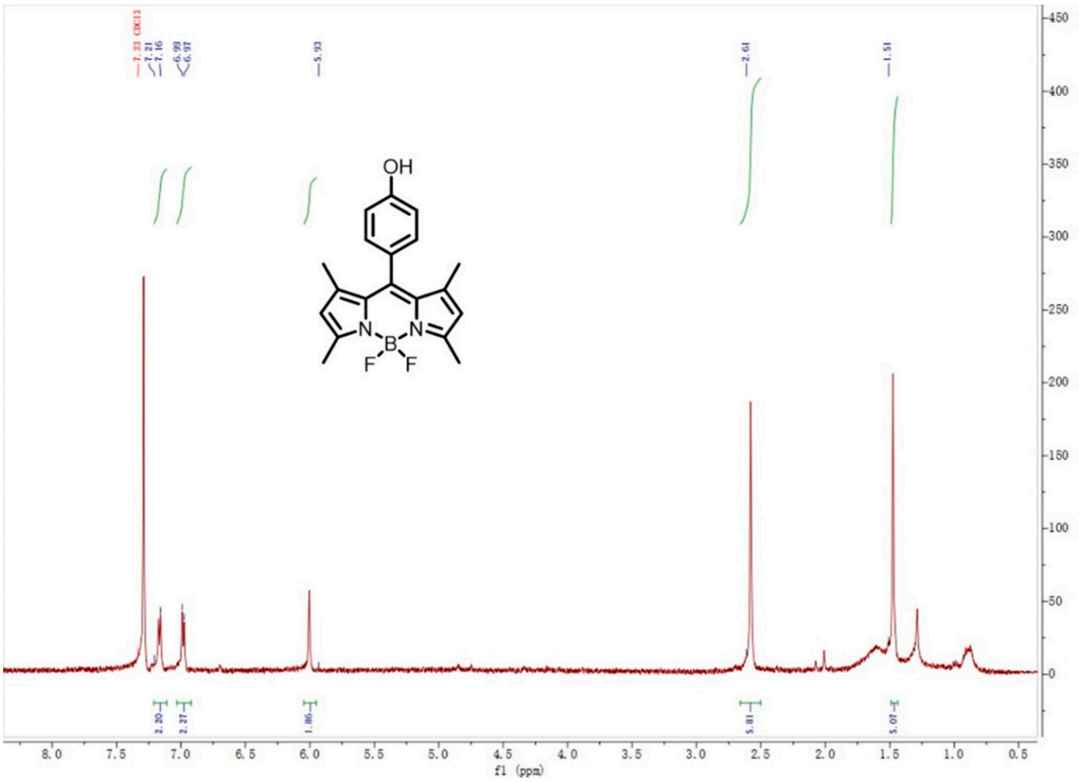

Figure S2. ^{1}H NMR spectrum of MOBDP in $CDCl_3$-d_4.

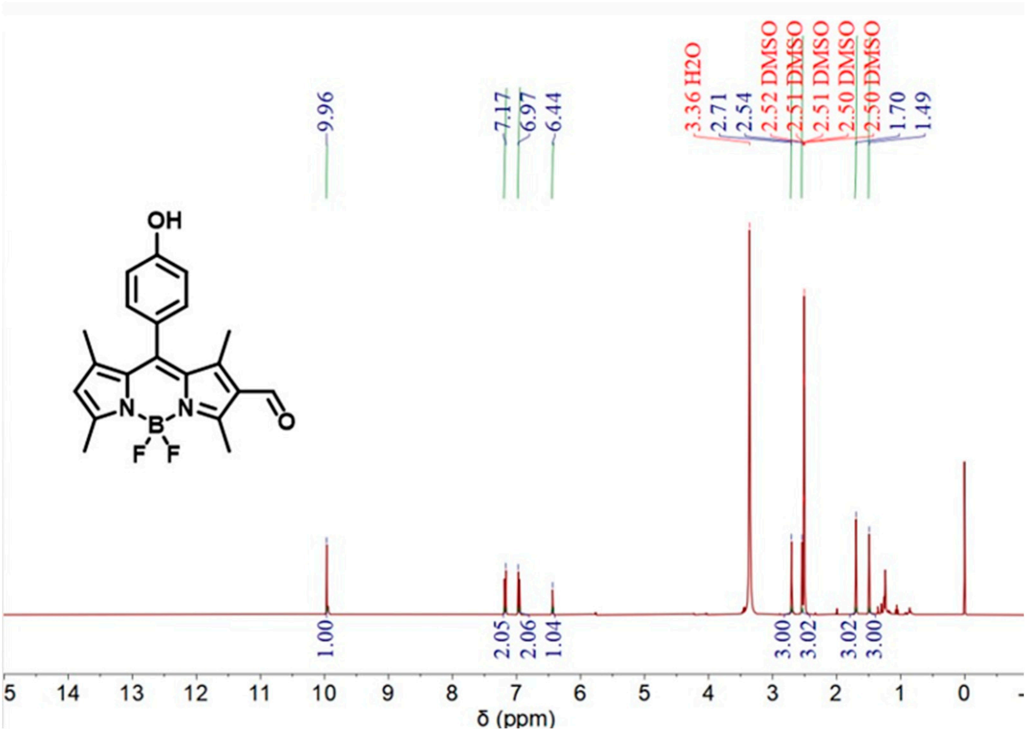

Figure S3. ^{1}H NMR spectrum of MOBDP-CHO in $CDCl_3$-d_4.

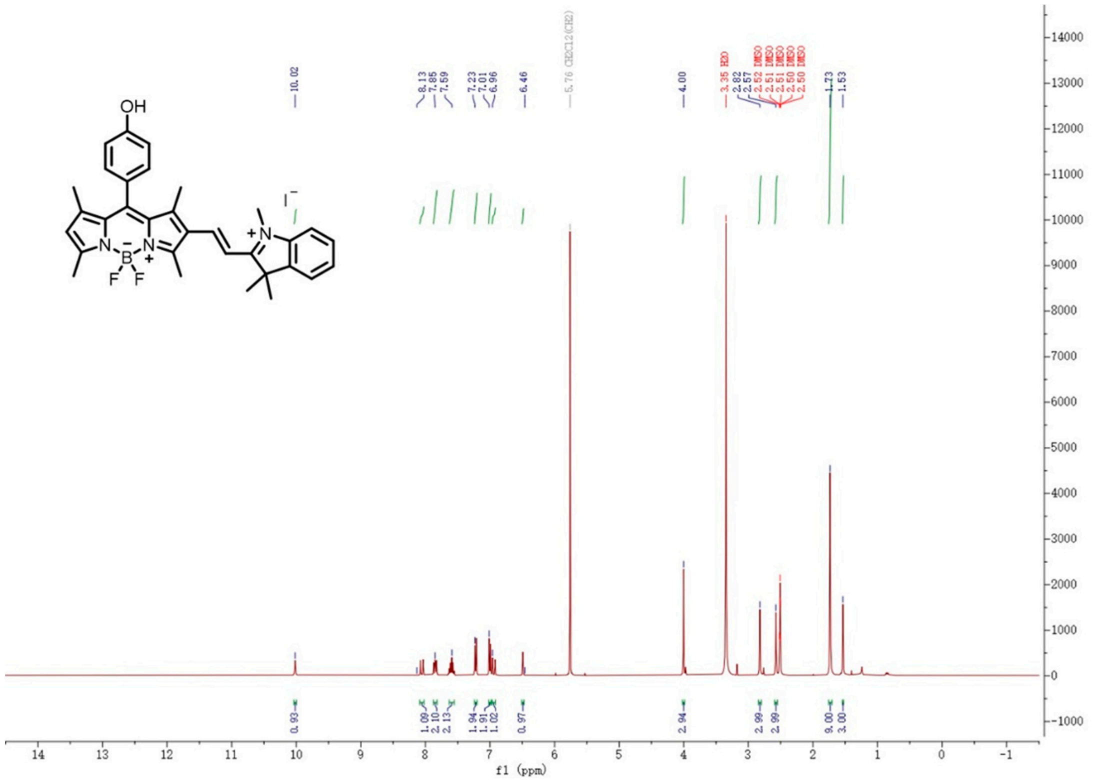

Figure S4. ^{1}H NMR spectrum of MOBDP-I in DMSO-d_6.

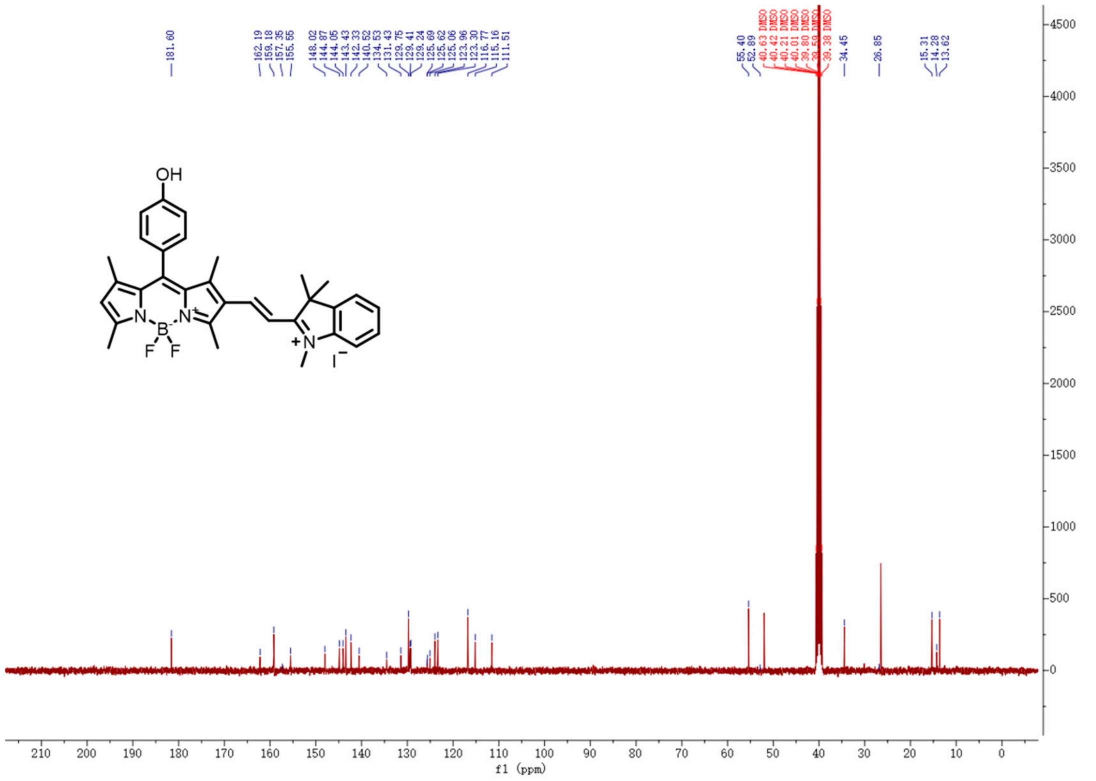

Figure S5. ^{13}C NMR spectrum of MOBDP-I in DMSO-d_6.

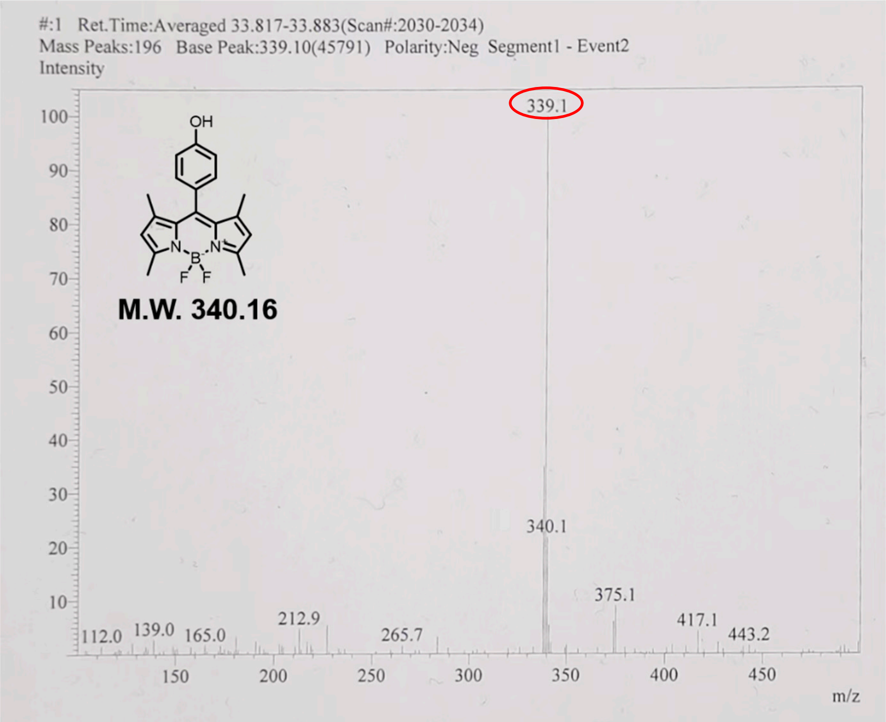

Figure S6. The mass spectrum (ESI) of MOBDP.

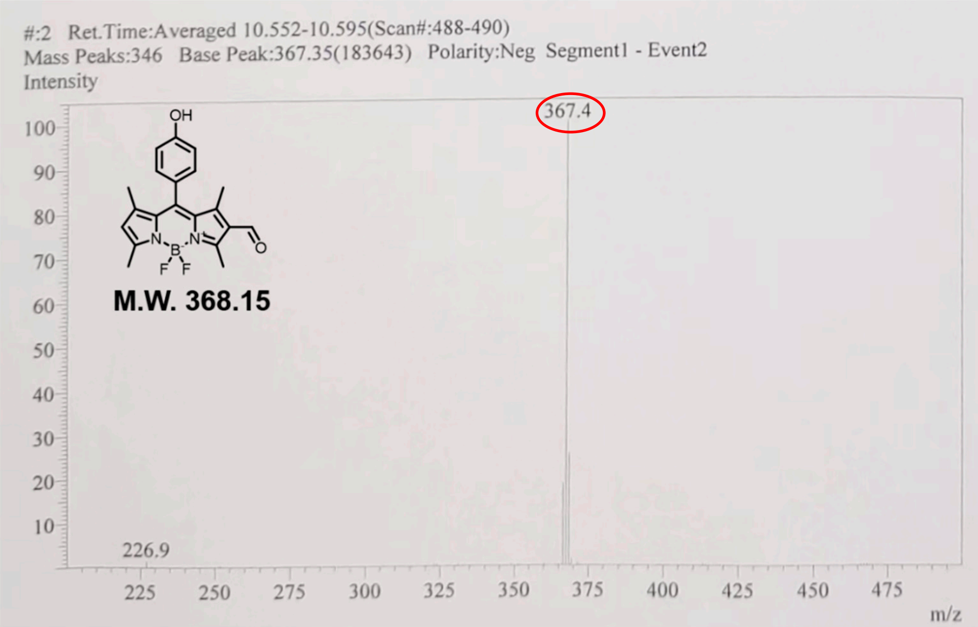

Figure S7. The mass spectrum (ESI) of MOBDP-CHO.

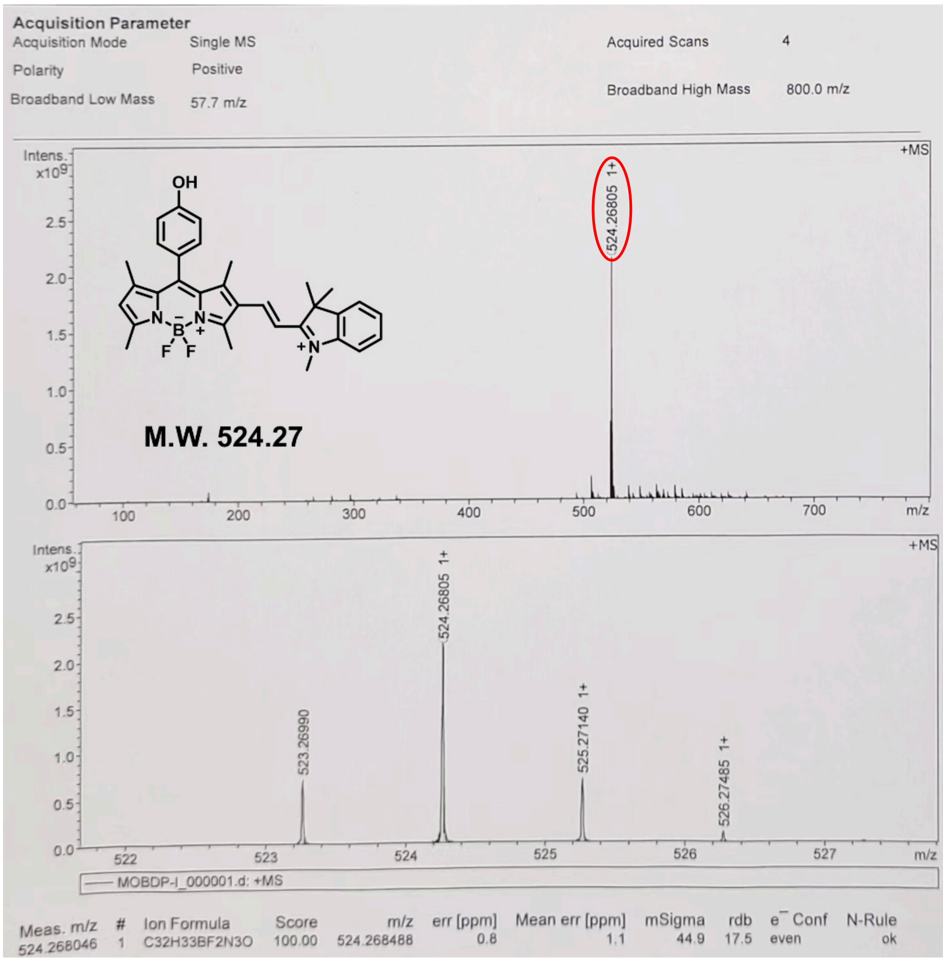

Figure S8. The mass spectrum (HRMS) of MOBDP-I. HRMS (ESI, positive) m/z. calcd for $C_{32}H_{33}BF_2N_3O^+$ ($[M]^+$): 524.2679; found: 524.2680.

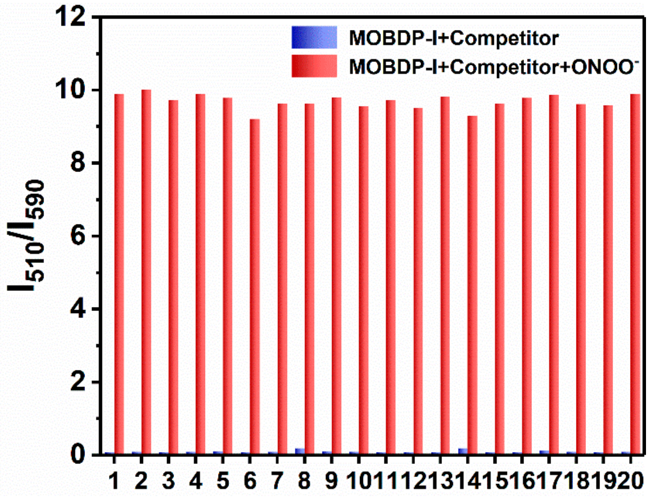

Figure S9. The fluorescence response intensity (I_{510}/I_{596}) of MOBDP-I to various analytes (50 μM) and $ONOO^-$ (10 μM) + analytes (50 μM). Analytes (1-20): $^1O_2$, ClO^-, H_2O_2, ROO^-, $\cdot OH$, NO_3^-, NO_2^-, H_2S, SO_3^{2-}, HSO_3^-, GSH, Cys, K^+, Na^+, Ca^{2+}, Mg^{2+}, Zn^{2+}, Cu^{2+}, Ni^{2+}, Co^{2+}.

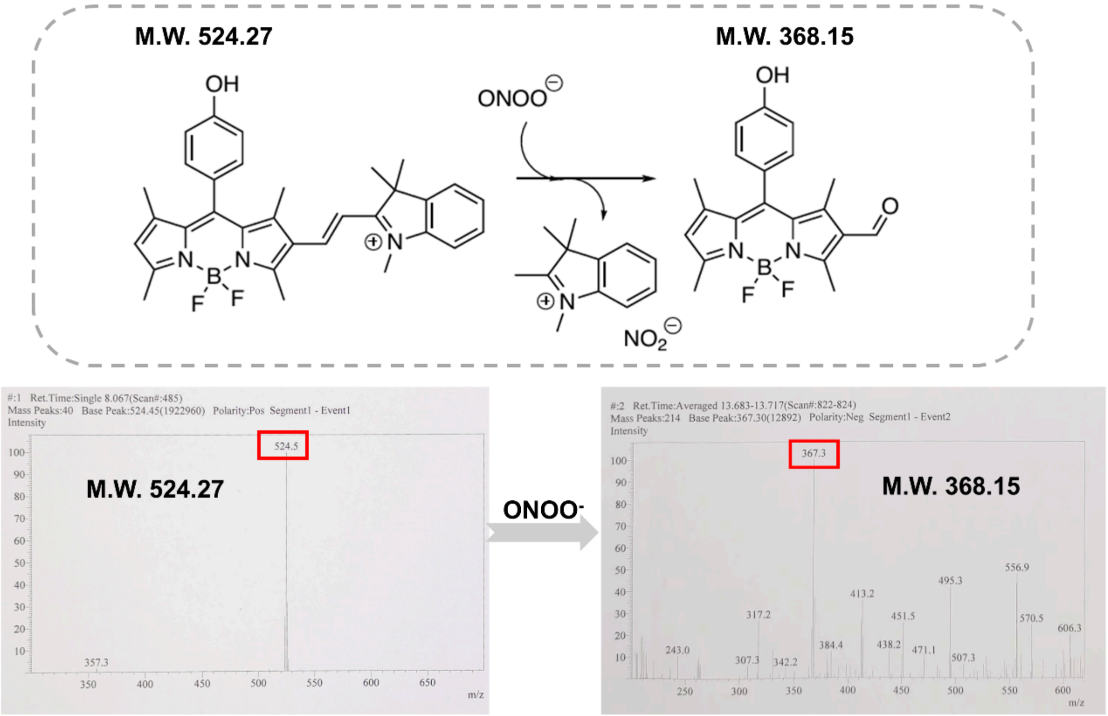

Figure S10. The reaction mechanism of MOBDP-I with ONOO$^-$ and mass spectra (ESI) of MOBDP-I and MOBDP-CHO. Note that MOBDP-I was analyzed in positive ion mode. The detected charged species was $[M]^+$, as itself is a cation. The product MOBDP-CHO was analyzed in negative ion mode. The detected charged species should be $[M-H]^-$.

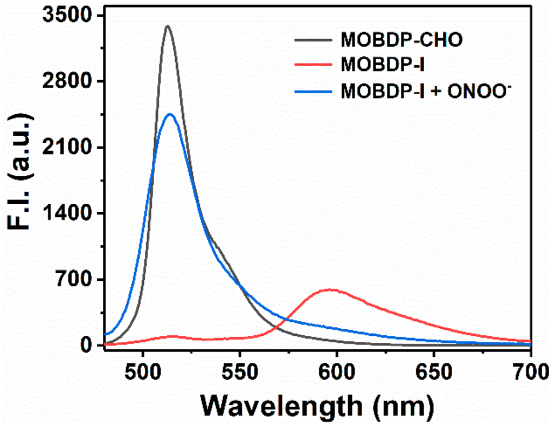

Figure S11. Fluorescence spectra of MOBDP-CHO (10 μM), MOBDP-I (10 μM) and MOBDP-I (10 μM) in response to ONOO$^-$ (10 μM) in PBS (pH = 7.4)

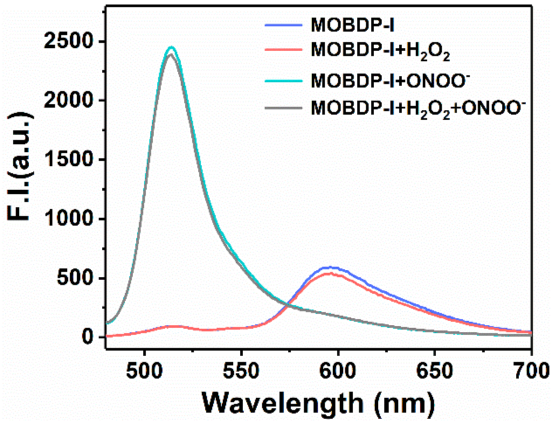

Figure S12. Fluorescence spectra of MOBDP-I (10 μM), MOBDP-I (10 μM) + H_2O_2 (50 μM), MOBDP-I (10 μM) + $ONOO^-$ (10 μM) and MOBDP-I (10 μM) + H_2O_2 (50 μM) + $ONOO^-$ (10 μM).

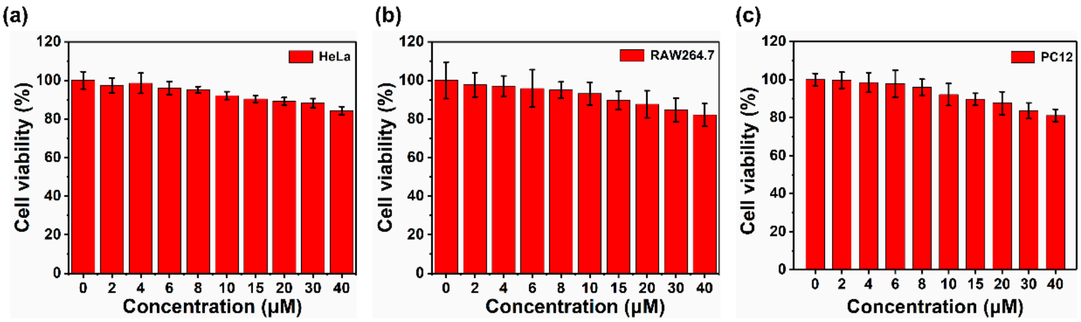

Figure S13. MTT assay of (a) HeLa cells, (b) RAW264.7 cells and (c) PC12 cells in the presence of different concentrations of MOBDP-I (0 ~ 40 μM). The error bars represent ± standard deviation (SD) (n = 3).

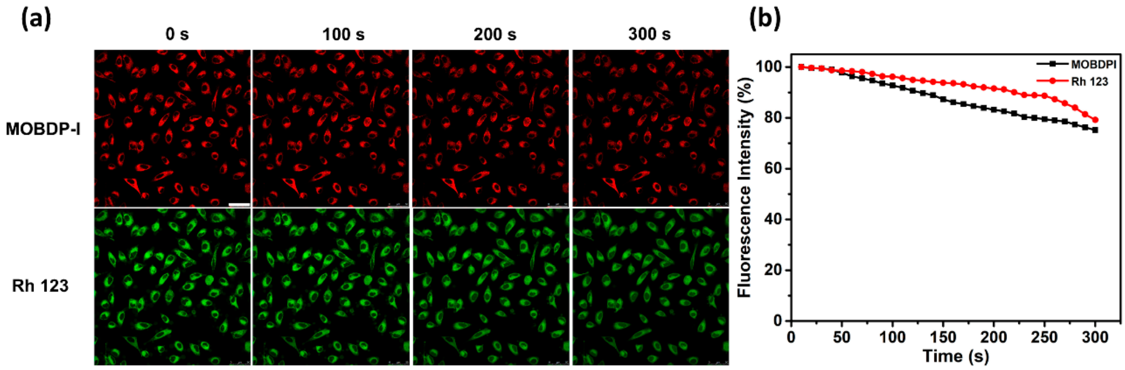

Figure S14. (a) Photostability of MOBDP-I (10 μM) and Rhodamine 123 (5 μM) incubated with HeLa cells, the signal intensity varies with the laser bleaching time. (Red channel: λ_{ex} =543 nm, λ_{em} = 580 – 650 nm. Green channel: λ_{ex} = 488 nm, λ_{em} = 510 - 540 nm, scale bar = 50 μm). (b) Normalized intensity profiles of average intracellular signals calculated from the confocal fluorescence images in (a) by applying Image*J*.

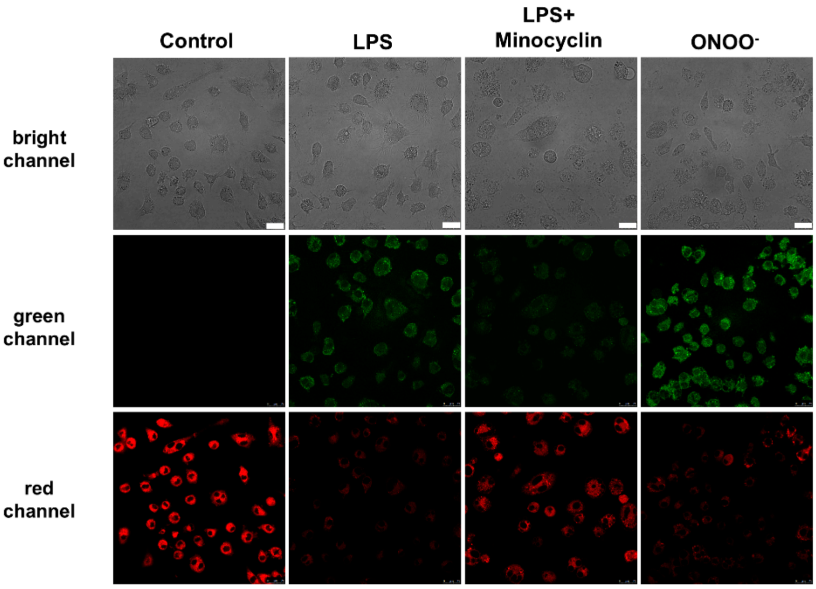

Figure S15. Confocal fluorescence imaging of $ONOO^-$ in RAW264.7 cells using the probe MOBDP-I (10 μM, 30 min). Control group: MOBDP-I only; LPS group: LPS (1 μg/mL, 6 h) then MOBDP-I; LPS + Minocycline group: LPS (1 μg/mL, 6 h), minocycline (300 μM, 2 h), then MOBDP-I; $ONOO^-$ group: exogenous $ONOO^-$ (10 μM, 30 min), then MOBDP-I. Green channel: λ_{ex} = 488 nm, λ_{em} = 510 - 540 nm. Red channel: λ_{ex} = 543 nm, λ_{em} = 600 - 650 nm, scale bar: 75 μm.

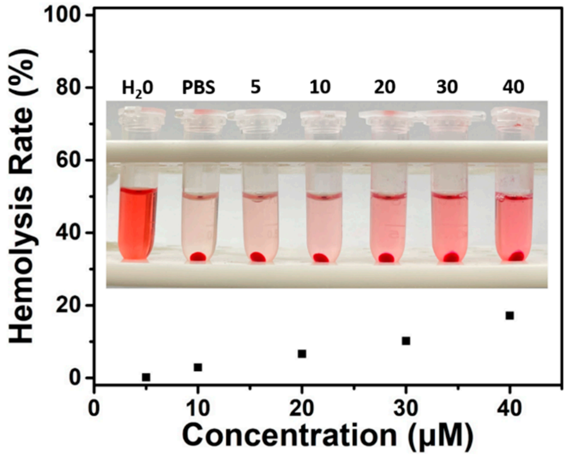

Figure S16. Hemolysis rate at different concentrations of MOBDP-I (0 - 40 μM).

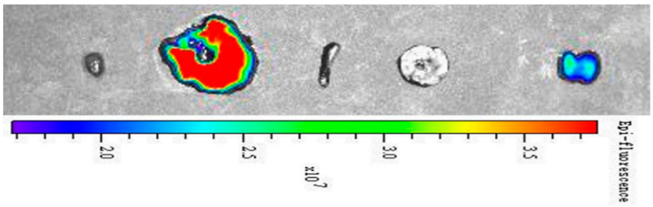

Figure S17. Fluorescence imaging in the major organs (heart, liver, spleen, lung, and kidney) isolated from mice.

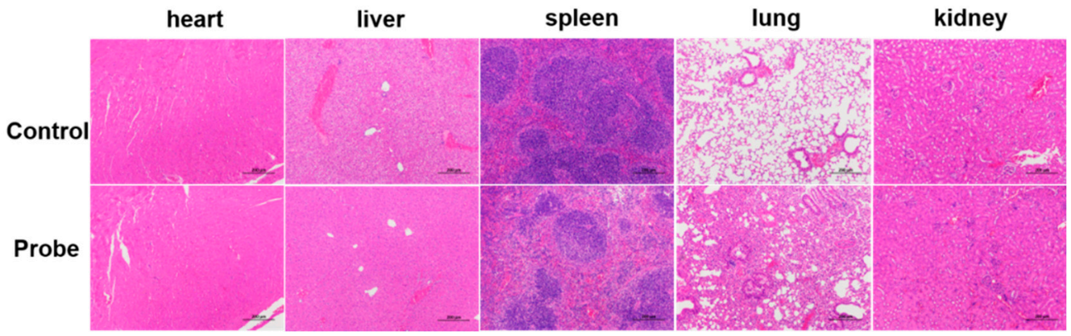

Figure S18. Hematoxylin and eosin (H&E) staining of major organs (heart, liver, spleen, lung, and kidney) collected from mice.

Table S1. Comparison of the proposed probe with other reported fluorescence probes for detecting peroxynitrite

No.	Probe	$\lambda_{ex}/\lambda_{em}$ (nm)	SNR (Fold)	LOD	Response time	Biological applications	Ref
**1**	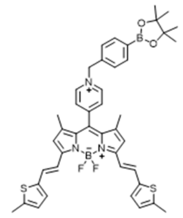	620/694 nm	66	120 nM	2 min	Imaging in cells and mouse peritonitis	**[4]**
**2**	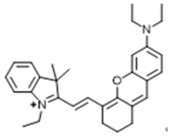	360/487 nm<br>710/742 nm	436	170 nM	150 s	Imaging in cells and mouse peritonitis	**[5]**
**3**	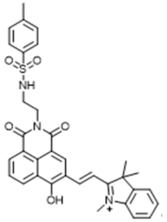	420/527 and 634 nm	22	930 nM	3 min	Imaging in cells and zebrafish	**[6]**
**4**	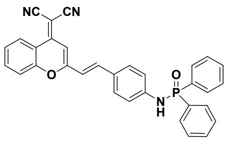	512/685 nm	4.5	96 nM	10 min	Imaging in cell and mice epilepsy model	**[7]**

**5**	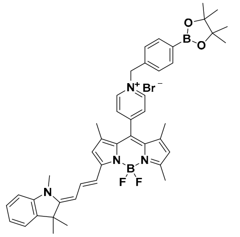	660/740 nm	247	50 nM	15 s	Imaging in cell and mice epilepsy model	**[8]**
**6**	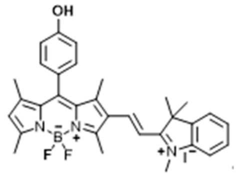	460/510 and 590 nm	44	9.6 nM	1 s	Imaging in cell and mouse models of rheumatoid arthritis, peritonitis and brain inflammation	**This work**

References

1. Xu, W.; Yang, Q.; Zeng, J.; Tan, L.; Zhou, L.; Peng, L.; Zhou, Y.; Xie, C.; Luo, K.; Zhang, Z. A biomarker ($ONOO^-$)-activated multicolor fluorescent probe for early detection and assessment of arthritis. *Sens. Actuators B Chem.* **2022**, *359*, doi:10.1016/j.snb.2022.131565.
2. Wang, Z.; Wang, W.; Wang, P.; Song, X.; Mao, Z.; Liu, Z. Highly Sensitive Near-Infrared Imaging of Peroxynitrite Fluxes in Inflammation Progress. *Anal. Chem.* **2021**, *93*, 3035-3041, doi:10.1021/acs.analchem.0c05118.
3. Sun, Y.; Tang, X.; Li, X.; Kong, X.; Tian, M.; Wang, Y.; Dong, B. PET-ESIPT-based fluorescent probes for revealing the fluctuation of peroxynitrite ($ONOO^-$) in living cells, zebrafishes and brain tissues. *Sens. Actuators B Chem.* **2022**, *353*, doi:10.1016/j.snb.2021.131121.
4. Qin, S.; Ran, Y.; He, Y.; Lu, X.; Wang, J.; Zhao, W.; Zhang, J. Near-Infrared Fluorescence Probe for Visualizing Fluctuations of Peroxynitrite in Living Cells and Inflammatory Mouse Models. *Chemosensors* **2023**, *11*, doi:10.3390/chemosensors11060316.
5. Zhou, D.Y.; Li, Y.; Jiang, W.L.; Tian, Y.; Fei, J.; Li, C.Y. A ratiometric fluorescent probe for peroxynitrite prepared by de novo synthesis and its application in assessing the mitochondrial oxidative stress status in cells and in vivo. *Chem Commun (Camb)* **2018**, *54*, 11590-11593, doi:10.1039/c8cc07389a.
6. Liu, Y.; Ma, Y.; Lin, W. Construction of a bi-functional ratiometric fluorescent probe for detection of endoplasmic reticulum viscosity and $ONOO^-$ in cells and zebrafish. *Sens. Actuators B Chem.* **2022**, *373*, doi:10.1016/j.snb.2022.132742.
7. Luo, X.; Cheng, Z.; Wang, R.; Yu, F. Indication of Dynamic Peroxynitrite Fluctuations in the Rat Epilepsy Model with a Near-Infrared Two-Photon Fluorescent Probe. *Anal. Chem.* **2021**, *93*, 2490-2499, doi:10.1021/acs.analchem.0c04529.
8. Han, R.; Ma, X.; Wang, J.; Zhang, B.; Ruan, M.; Jiao, J.; Zhao, W.; Zhang, J. Novel BODIPY-based NIR fluorescent probe with appropriate Stokes shift for diagnosis and treatment evaluation of epilepsy via imaging $ONOO^-$ fluctuation. *Sens. Actuators B Chem.* **2025**, *423*, doi:10.1016/j.snb.2024.136766.
